# Complex Focal Pain Syndrome: An Unusual Variant of Complex Regional Pain Syndrome

**DOI:** 10.7759/cureus.9510

**Published:** 2020-08-01

**Authors:** Roger Bui, Jason Coffman, Andrew Berry, John J Faillace

**Affiliations:** 1 Orthopaedic Surgery and Rehabilitation, The University of Texas Medical Branch, Galveston, USA; 2 Plastic and Reconstructive Surgery, The University of Texas Medical Branch, Galveston, USA

**Keywords:** reflex sympathetic dystrophy, complex regional pain syndrome, pain, crush injury, trauma, neuropathic pain, causalgia

## Abstract

Complex regional pain syndrome (CRPS) is a chronic neuropathic pain condition that is often overlooked by clinicians and typically occurs within an entire limb. There is considerable clinical variability in presentation among patients with CRPS. We report a case of extremely focal CRPS localized to the left small finger (LSF) following crush injury. A 48-year-old right-handed male presented with LSF stiffness and severe pain of three months’ duration following crush injury. He endorsed severe allodynia and minimal flexion at the proximal interphalangeal and distal interphalangeal joints of the LSF. Physical examination was significant for overt shininess and edema isolated to the LSF. X-ray performed at the time of injury and three months after were devoid of any fracture or dislocation. Chronic focal pain syndrome (CFPS) may be a subset of CRPS that has yet to be documented in the literature.

## Introduction

Complex regional pain syndrome (CRPS) is a chronic neuropathic pain condition characterized by disarray of sensory, autonomic, trophic, and motor systems [1]. The pain can be further classified into type I, which results from soft-tissue or bone injury, or type II, a consequence of nerve injury. While the pathophysiology of CRPS has been elusive for several years now, three major pathways have been postulated: aberrant inflammatory cascade, vasomotor dysfunction, and maladaptive neuroplasticity. Consequently, the literature supports the multifactorial nature of CRPS that is closely associated with maladaptive host responses to tissue insult.

Considerable clinical variability exists among patients with CRPS; however, they typically present after minor to moderate tissue injury, such as a fracture. A prospective study performed by Jellad et al. reported an incidence of CRPS as high as 32.2% among patients with distal radius fracture [2]. Frequently, CRPS occurs between the third and fourth week of cast removal. Acutely, patients may present with an erythematous and edematous extremity. Furthermore, the patient may exhibit hypersensitivity to stimuli in the form of allodynia or hyperalgesia. Conversely, the patient may have negative sensory symptoms: hypoesthesia, hypoalgesia, and hypothermesthesia [3-5]. Other manifestations include autonomic changes (vasomotor abnormalities), trophic changes (skin changes and bone atrophy), and motor changes (paralysis, weakness, dystonia) [6]. There tends to be marked voluntary motor impairment as the disorder persists; movement of the extremity may exacerbate the signs and symptoms. The diagnosis is made clinically, and there are no confirmatory tests that can be performed. Utilization of the Orlando criteria or the modified version, the Budapest criteria, yields the diagnosis of CRPS. We report a unique case of a patient who sustained crush injury to the left small finger (LSF) three months prior and presented with focal CRPS of the LSF. X-ray performed at the time of injury showed no fracture.

## Case presentation

**Figure 1:**
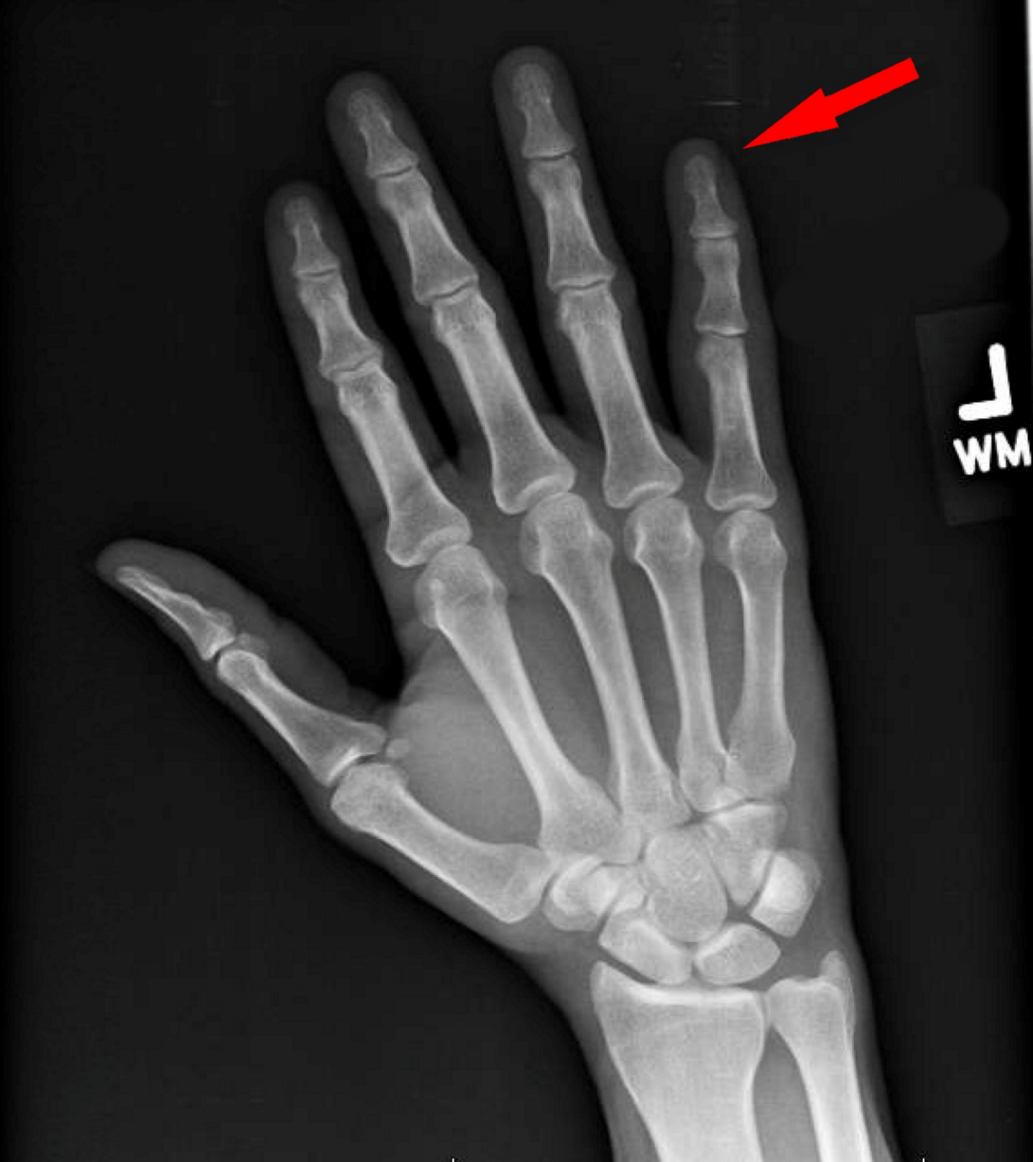
X-ray dorsal view of the left hand immediately following crush injury.

A 48-year-old, right-handed male presented to clinic with the complaint of LSF stiffness and severe pain of three months’ duration. He stated that he “fractured” his LSF by crushing it in a door three months previously. X-ray performed at that time showed no acute fracture, dislocation, or soft-tissue changes (Figure 1). Conservative management was sought with an ulnar gutter splint. The patient took it off himself after one month due to lack of improvement. He had been undergoing hand therapy and rehabilitation for approximately three weeks and endorsed minimal functional improvement with the therapy.

Upon physical examination, there was overt shininess and edema isolated to the LSF. The LSF was severely tender to palpation. There was considerable hypersensitivity to light touch at the LSF. Motor strength was intact to intrinsic and extrinsic muscles. There was minimal active flexion at the proximal interphalangeal (PIP) and distal interphalangeal (DIP) joints of the LSF. However, there was a small amount of preserved flexion at the DIP joint. Brisk capillary refill was present in that extremity.

**Figure 2:**
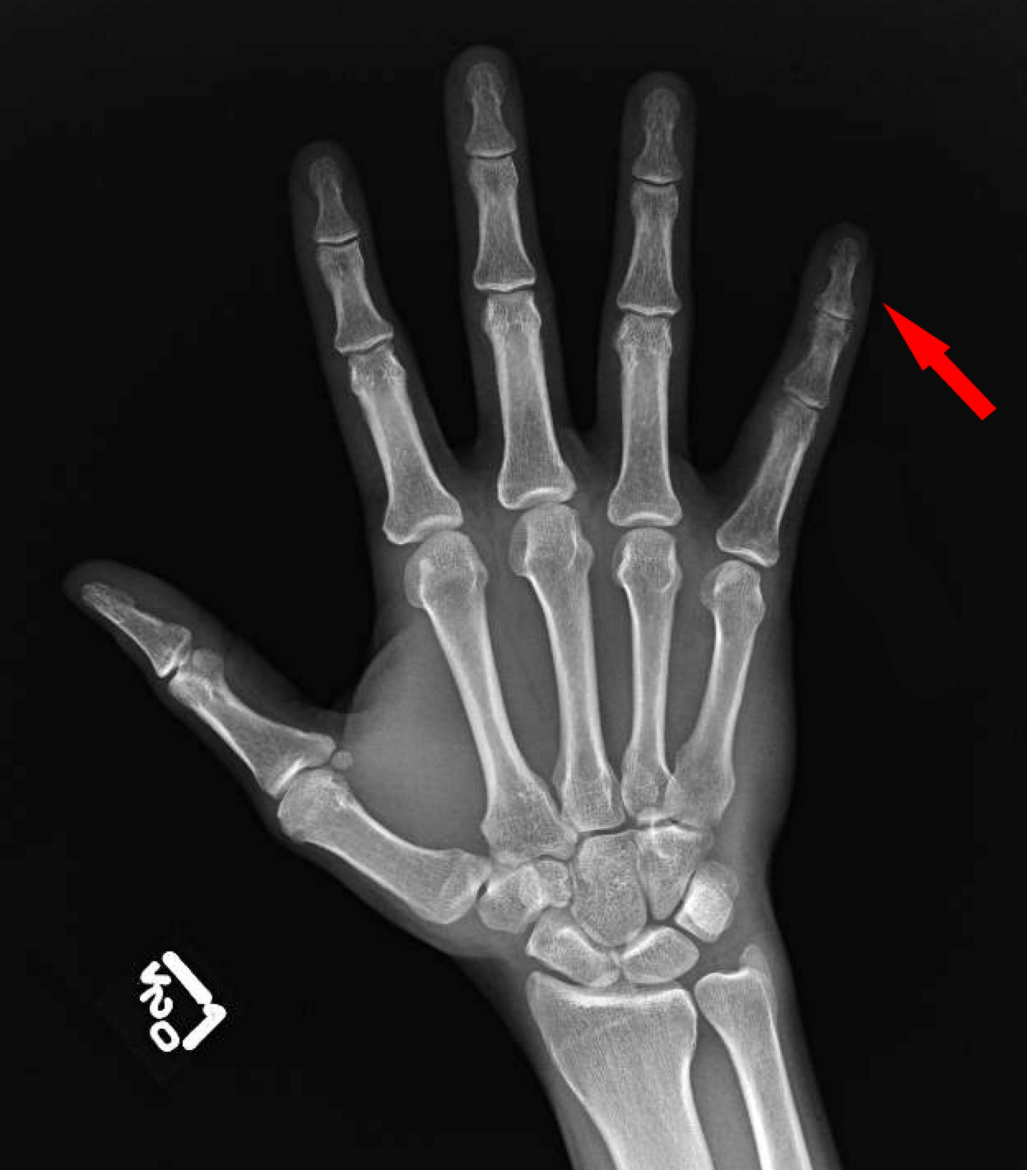
X-ray dorsal view of the left hand three months following crush injury.

A repeat X-ray revealed focal osteopenia within the small finger phalanges, likely related to disuse (Figure 2). Mild soft-tissue swelling within the small finger was also noted. The imaging was devoid of any acute or chronic osseous changes that could explain his symptomology. Ultimately, the patient’s treatment plan for CRPS included recommendations to initiate gabapentin, duloxetine, and high-dose vitamin C. The crux of treatment also included desensitization therapy, range-of-motion therapy, and activity modification to minimize the pain. The patient had followed up in clinic three months later endorsing only modest improvement in active flexion at the PIP and DIP joints of LSF. Hypersensitivity to light touch and edema persisted despite conservative management. The patient was ultimately lost to follow-up six months after his crush injury. 

## Discussion

Literature documenting an extremely focal chronic pain syndrome is sparse. Typically, following traumatic injury to an extremity, or even a portion of that extremity, the hallmark signs and symptoms of CRPS ensue; the neuropathic pain that manifests as allodynia and hyperalgesia classically involves a whole region of that extremity, as opposed to a single digit. Over time, clinical features have a proclivity to disseminate proximally and may even emerge on the opposite or ipsilateral limb [7-9]. The incidence of CRPS is unclear, but a population-based study has yielded approximately five cases per 100,000 person-years in the United States [5]. The arm is generally affected in 60% of the cases and the leg in 40% [10]. Fractures (45%), sprains (18%), and surgery (12%) are the most frequently reported triggering events of CRPS. Although the patient in this case did not sustain any discernible fractures, sprains, or undergo elective surgeries, the blunt trauma inflicted on his finger was likely the inciting event.

CRPS is a difficult condition to treat due to the clinical heterogeneity and multifactorial nature of the syndrome. The areas of treatment include emphasis on physical activity, desensitization, and normalization of sympathetic tone modalities that help facilitate functional restoration. Occupational and physical therapy are paramount to the process of functional restoration. Additional interventions include pharmacotherapy involving non-steroidal anti-inflammatory drugs (NSAIDs), corticosteroids, cation channel blockers (gabapentin, pregabalin, carbamazepine), heterocyclic antidepressants, N-methyl-D-aspartate (NDMA) receptor antagonists, and α-adrenergic antagonists [11]. Moreover, this case illustrates just how tenacious and how much of a functional detriment CRPS can be despite consistent physical rehabilitation prompt pharmacotherapy implementation. Despite three months of consistent physical therapy as well as treatment with duloxetine and gabapentin, only modest improvement was noted in range of motion and hypersensitivity. Escalation of treatment may include various interventional therapies, such as sympathetic nerve blocks, intravenous (IV) regional techniques, spinal infusions, neurolytic sympathetic blockade, and implantable therapies (neurostimulators and intrathecal pumps) [11]. The utility of psychologic intervention remains equivocal due to the paucity of prospective studies examining psychological factors in the onset and maintenance of CRPS.

Based on the patient’s history and physical exam findings, several Budapest diagnostic criteria were met: continuing pain disproportional to the inciting event, allodynia, edema, sweating asymmetry, and decreased range of motion. Additionally, there are no other diagnoses that would better explain the patient’s signs and symptoms. This case is unique in that it that fulfills both the Orlando and Budapest diagnostic criteria. There should be a high index of suspicion for CRPS, even if it is limited to a focal area. The diagnosis was further bolstered by the unremarkable X-ray findings and lack of any comorbidities that could explain his intense LSF pain.

## Conclusions

This case report highlights the importance of keeping CRPS in consideration for patients with a history of trauma to an extremity and subsequent hallmark signs and symptoms of the pain condition (hypersensitivity to pain, vasomotor, trophic, and autonomic disturbances), even if isolated to a focal area. Moreover, the case emphasizes how difficult it is to treat CRPS despite employing interventions that are supported in the literature. We propose a subset of CRPS, called chronic focal pain syndrome (CFPS), be used to describe this population of patients and raise awareness of the condition.
